# Supplementary material for: Assessing the reliability of abdominal adiposity measurements by transabdominal ultrasound in first trimester pregnancies with sonographers of varying experience levels
Source: PLoS One. 2025 Jun 26;20(6):e0326949. doi: 10.1371/journal.pone.0326949 (PMC12200860; doi:10.1371/journal.pone.0326949)
Supplement: Attachment — Submitted filename: Review_OBSTETRICS ARTICLE.pdf [file pone.0326949.s003.pdf]

REVIEW_OBSTETRICS ARTICLE

ASSESSING THE RELIABILTY OF ABDOMINAL ADIPOSITY MEASUREMENTS BY TRANSABDOMINAL ULTRASOUND IN FIRST TRIMESTER PREGNACIES WITH SONOGRAPHERS OF VARYING EXPERIENCE LEVELS

Summary

This study is significant and has relevance in maintaining the health of both the mother and fetus. The aim and objective of the study were achieved through the different methods, results and the discussion. The study targeted the right subjects and the tools were non-invasive which made the methods more attractive. And elicited low or no discomfort to the pregnant women during their applications.

Abstract

Can the reason why these tools were considered be incorporated in the abstract?

Yes, it is to measure the abdominal adiposity in pregnancy. But why? Line 28…. Let the correlation between the measurement of the abdominal adiposity and gestational diabetes mellitus be incorporated.

Introduction

Line 66…. Gave the reasons why this study was conducted, however, I believe this tool would be used in conjunction with other known methods in determining/diagnosing gestational diabetes mellitus.

Materials and Methods

Line 119 – 122…. What informed you of these 3 locations for the measurements?

Results

Line 153 – 155…. The probability that the procedure and results would be affected by experience of the operators was expected to be high as rightly indicated in this study. However, line 158, interestedly showed that VFT was not affected by experience.

Discussion

Line 181… this sentence should be reworded. 'For Thai population, one study found similar results to ours with high intraobserver reliability (ICC 0.96)' …. It could have be written as 'our result was similar to a previous study which had a high intraobserver reliability' (ICC 0.96). My understanding is that you compare new study with previous studies.

Figures and Tables: They were simple, self-explanatory, easy to the eye and well labelled.
