# Supplementary material for: Assessing the reliability of abdominal adiposity measurements by transabdominal ultrasound in first trimester pregnancies with sonographers of varying experience levels
Source: PLoS One. 2025 Jun 26;20(6):e0326949. doi: 10.1371/journal.pone.0326949 (PMC12200860; doi:10.1371/journal.pone.0326949)
Supplement: Attachment — Submitted filename: Response to ReviewersAll.docx [file pone.0326949.s004.docx]

**Response to Reviewers**

**Reviewer #1**

**Comment 1:** Line 109-110: The authors mentioned exclusion criteria -- As this is a study about the reliability, I don't see why the authors had to exclude these women. If the study is a part of other studies that require these exclusion criteria, the authors should clarify.

**Reply 1:** Thank you for your comment. This study is part of a larger prospective study evaluating the predictive ability of first-trimester ultrasound measurements of maternal adiposity for predicting gestational diabetes mellitus. Therefore, specific exclusion criteria were applied to minimize potential confounding effects on outcomes.

**Change in the text:** No changes were made, but we have clarified this point in our response.

**Comment 2:** Line 118: "(operator 1 and 2, operator 1 and 3, and operator 2 and 3)" gives meaning of each participant receive U/S from 3 sonographers, the authors might consider change to "(operator 1 and 2, operator 1 and 3, 'or' operator 2 and 3)."

**Reply 2:** We have revised the wording for improved clarity.

**Change in the text:** Updated in the Materials and Methods section (lines 118).

**Comment 3:** The authors might consider mention about probe pressure during the measurement.

**Reply 3:** We appreciate this suggestion. We have added a statement indicating that ultrasound measurements were taken with care to avoid excessive probe pressure, which could artificially compress abdominal tissue.

**Change in the text:** Added this detail in the Materials and Methods section (lines 123-124).

**Comment 4:** The authors might consider clarify more about the position of the U/S probe for the "cervix-placenta view"

**Reply 4:** Thank you for your comment. We have included additional details regarding the ultrasound probe positioning for the "cervix-placenta view."

**Change in the text:** Added in Table 1.

**Comment 5:** Table 2 - misspelling "Wight circumference"

**Reply 5:** We appreciate your careful review. The spelling error has been corrected.

**Change in the text:** Updated in Table 2.

**Comment 6:** The method section mentioned Bland-Altman plots; however, I could not find the plot in the result section. The authors might rephrase the method section to make it align.

**Reply 6:** Thank you for pointing this out. The Bland-Altman plots were used initially to assess reliability, followed by modifications to establish limits of agreement, which represent the variability range in differences and indicate test-retest reliability for 95% of the population. We have revised the text for clarity.

**Change in the text:** Updated in Materials and Methods section (lines 133-136).

**Reviewer # 2**

**Comment for introduction part:**

The authors refer to in-text citations using "ref" before the bracketed numbers (e.g., "ref 1-2"), which is unnecessary.

**Reply:** We agree and have corrected all in-text citations to follow the appropriate format using Arabic numerals in square brackets.

**Change in the text:** Updated throughout the manuscript.

**Comment for materials and methods part:**

What advised the choice of statistical analysis used by the authors?

**Reply:** The statistical analysis used in this study was advised and assisted by Dr. Alan Geater, Ph.D., a clinical epidemiology consultant from the Epidemiology Unit, Faculty of Medicine, Prince of Songkla University. This contribution is acknowledged in the Acknowledgements section.

**Change in the text:** No changes needed, as this is mentioned in Acknowledgements.

Was the data tested for normality before deciding on the statistical analysis?

**Reply:** Yes, normality testing was performed before statistical analysis to ensure the appropriateness of the methods used.

**Change in the text:** No changes needed, but we confirm this in our response.

Did the authors run a positive and negative predictive values test for the study to determine the reliability of Ultrasound in adiposity biometry?

**Reply:** Our objective was to evaluate the reliability of sonographic measurements of abdominal adiposity during the first trimester of pregnancy, not to assess their predictive value for other outcomes. Therefore, we did not perform positive or negative predictive value tests in this study.

**Change in the text:** No changes needed, but we confirm this in our response.

**Comment for results part:**

The numbering of tables starts at Table 2. The authors should ensure Table 1 presents demographic characteristics, while other information could be better presented as figures.

**Reply:** Thank you for your suggestion. In our opinion, Table 1, which describes the standard protocol for measuring each parameter, provides necessary details and is best suited for a tabular format.

**Change in the text:** No changes were made.

What advised the selection of only 90 participants?

**Reply:** Our objective was to evaluate intraobserver variability among operators with different experience levels. Each participant was independently scanned by two operators in the following pairings: (operator 1 and 2, operator 1 and 3, or operator 2 and 3). A sample size of 30 participants per operator pair was deemed sufficient to test reliability.

**Change in the text:** No changes were made, but we clarify this point in our response.

The tables are not self-explanatory, the lines within the tables should be removed!

**Reply:** Thank you for your suggestion. We have reformatted the tables accordingly.

**Change in the text:** Updated table formatting.

**Comment for discussion part:**

The authors refer to in-text citations using both initials and surnames (e.g., "NJ Kennedy et al." and "Nassr AA et al."). This should be corrected to "Kennedy et al." and "Nassr et al."

**Reply:** We appreciate your attention to detail. The in-text citations have been corrected accordingly.

**Change in the text:** Updated in the Discussion section (lines 206-207).

The discussion is somewhat narrow or shallow, can authors expand on the discussion please?

**Reply:** Thank you for your suggestion. As the main objective of this study was to evaluate the reliability of sonographic measurements of abdominal adiposity during the first trimester of pregnancy, we found limited previous studies in this field. While our discussion may appear concise, we believe this study provides a novel foundation for further global research in this area.

**Change in the text:** No changes were made.
