# Supplementary material for: Assessing the reliability of abdominal adiposity measurements by transabdominal ultrasound in first trimester pregnancies with sonographers of varying experience levels
Source: PLoS One. 2025 Jun 26;20(6):e0326949. doi: 10.1371/journal.pone.0326949 (PMC12200860; doi:10.1371/journal.pone.0326949)
Supplement: Attachment — Submitted filename: Response to Reviewers.docx [file pone.0326949.s005.docx]

**Response to Reviewers**

**Reviewer #3**

**Comment 1:** Why did you select the first trimester? Was it based on previous reports? We know that fat deposits increase through the second and third trimester, and these are the periods where pregnant women develop the actual insulin resistance.

**Response:** We selected the first trimester because it represents a baseline period before the onset of significant insulin resistance in pregnancy. Early identification of central adiposity may offer predictive insights before metabolic changes occur. Additionally, conducting assessments in the first trimester helps reduce potential confounding from physiological changes occurring later in pregnancy.

**Change in the text:** We have added justification in the Introduction section (Lines 107-111).

**Comment 2:** The second concern is the age. If you included younger women at 20th for example, this can remove the age factor of fat deposition.

**Response:** Thank you for this point. Our inclusion criteria accepted a wide age range to reflect real-world pregnant populations.

**Change in the text:** No changes were made, but we have clarified this point in our response.

**Comment 3:** Why did you select different levels of trainees to do the abdominal scan? As a first report in the country, you should put a solid background for future research by conducting your study with expert hands.

**Response:** Our study aimed to evaluate the reliability of sonographic measurements across varying operator experience levels, as this reflects actual clinical scenarios, particularly in resource-limited settings. We now clarify this rationale in the Methods and Discussion sections.

**Change in the text:** No changes were made, but we have clarified this point in our response.

**Comment 4:** Why did you exclude hypertensive women? Hypertension alone has no impact on fat deposition unless related to obesity or diabetes.

**Response:** We excluded women with hypertension to minimize confounding effects, as chronic hypertension may independently influence vascular and metabolic status. Moreover, ACOG Practice Bulletin No. 190 identifies hypertension as a risk factor for gestational diabetes mellitus, supporting our decision to exclude such cases.

**Change in the text:** No changes were made, but we have clarified this point in our response.

**Reviewer #4**

**Comment 1:** Tables 3 and 4 are hard to interpret. Could a summary be used or move data to supplementary materials?

**Response:** Thank you for the suggestion. Tables 3 and 4 present key findings regarding intraobserver and interobserver variability, which directly relate to the primary objective of our study. Therefore, we believe they should remain in the main manuscript. However, we have added a concise summary of the main findings in the Results section (Lines 174-180).

**Change in the text:** No changes were made, but we have clarified this point in our response.

**Comment 2:** Findings on inter-sonographer variability do not stand on their own; suggest linking to broader study of validity or predictive value.

**Response:** We appreciate this valuable feedback. We have now clarified in the Introduction and Discussion that this study serves as a foundational methodological assessment of measurement reliability. Future studies will build on this by evaluating predictive value using standard diagnostic outcomes.

**Change in the text:** No changes were made, but we have clarified this point in our response.

**Reviewer #5**

**Comment 1 (Abstract):** Why were these tools considered? Line 28: Let the correlation between abdominal adiposity and GDM be incorporated.

**Response:** We revised the abstract to clearly indicate that the study was motivated by the potential association between abdominal adiposity and GDM risk.

**Change in the text:** Updated in the Abstract section (Lines 27-29).

**Comment 2 (Introduction):** Line 66: This tool would be used alongside other methods for diagnosing GDM.

**Response:** The section was revised to reflect that sonographic assessment of adiposity is a complementary tool, not a replacement for current GDM screening strategies.

**Change in the text:** Updated in the Introduction section (Lines 91-93 and 116-120).

**Comment 3 (Methods):** Lines 119–122: What informed you of the 3 locations for measurement?

**Response:** The three measurement sites were selected based on prior studies and anatomical landmarks representing distinct fat compartments.

**Change in the text:** We have added explanation and references in Materials and Methods (Lines 140) and Table 1.

**Comment 4 (Results):** Line 153 – 155: The probability that the procedure and results would be affected by experience of the operators was expected to be high as rightly indicated in this study. However, line 158, interestedly showed that VFT was not affected by experience.

**Response:** This observation is now more clearly emphasized in the Results section. We highlight that the ICC indicated excellent reliability across all adiposity parameters, with only the VFT measurement between the most and least experienced operators showing slightly lower, but still good, reliability (Lines 174-180).

**Change in the text:** No changes were made, but we have clarified this point in our response.

**Comment 5 (Discussion):** Line 181: Suggest rewording to compare clearly with previous studies.

**Response:** We revised the sentence to: “Our result was similar to a previous study which had a high intraobserver reliability (ICC 0.96).”

**Change in the text:** Updated in the Discussion section (Lines 202-203).

**Reviewer #6**

**Comment 1:** Line 83: “difference” should be “different”

**Response:** This has been corrected.

**Change in the text:** Updated in the Introduction section (Lines 109).

**Comment 2:** Line 90: Introduction suggests ultrasound is for GDM screening, but simple methods exist and obesity is not the only risk factor.

**Response:** We revised the Introduction to clarify that our aim is not to replace existing screening methods, but to explore the potential of ultrasound as a supportive tool for early risk identification based on adiposity.

**Change in the text:** Updated in the Introduction section (Lines 116-120).

**Comment 3:** Line 118: Operators are not equally distributed by experience.

**Response:** We acknowledge this and explain that our study design reflects real-world clinical practice, particularly in resource-limited settings, where varying levels of operator experience are common. Our study thus aimed to evaluate measurement reliability in such settings.

**Change in the text:** No changes were made, but we have clarified this point in our response.

**Comment 4:** Line 121: “Cervix-placenta view” needs explanation for how it measures VFT.

**Response:** Thank you for the comment. The cervix-placenta view (technique 2), as described in Table 1, was used only to measure SFT. VFT was measured using technique 1, at the level of the linea alba, 1–2.5 cm above the umbilicus (Lines 140-144).

**Change in the text:** No changes were made, but we have clarified this point in our response.

**Comment 5:** Line 190: Why focus only on operator reliability when prediction of GDM is emphasized in the Introduction?

**Response:** While the introduction highlights the potential association between abdominal adiposity and the risk of GDM, the primary objective of this study was to assess the reliability of sonographic measurements of abdominal adiposity in the first trimester. Assessing predictive value for GDM would require longitudinal follow-up, which are beyond the scope of this methodological assessment. We now clarify this point in the text.

**Change in the text:** No changes were made, but we have clarified this point in our response.
